# Infectious interface keratitis after Descemet membrane endothelial keratoplasty

**DOI:** 10.5935/0004-2749.20220059

**Published:** 2025-02-11

**Authors:** Nesrin Tutas Gunaydin, Burak Tanyıldız, Baran Kandemir, Saban Simsek

**Affiliations:** 1 Ophthalmology Department, University of Healty Sciences Dr. Lutfi Kırdar Kartal Training and Research Hospital, Istanbul, Turkey

**Keywords:** Corneal transplantation, Descemet membrane, Graft survival, Infections, Injections, Keratitis, Keratoplasty, penetrating, Visual acuity, Transplante de Córnea, Lâmina limitante posterior, Sobrevivência de enxerto, Infecções, Injeções, Ceratite, Ceratoplastia penetrante, Acuidade visual

## Abstract

**Purpose:**

To evaluate the clinical course and management of infectious interface keratitis after Descemet membrane endothelial keratoplasty.

**Methods:**

A total of 352 cases that had undergone Descemet membrane endothelial keratoplasty were retrospectively reviewed. Patients with infectious interface keratitis during follow-up were analyzed. The microbiological analyses, time to infection onset, clinical findings, follow-up duration, treatment, and post-treatment corrected distance visual acuity were recorded.

**Results:**

IIK was detected in eight eyes of eight cases. Three fungal and three bacterial pathogens were identified in all cases. All patients received medical treatment according to culture sensitivity. Antifungal treatment was initiated in two cases with no growth on culture, with a preliminary diagnosis of fungal interface keratitis. Intrastromal antifungal injections were performed in all patients with fungal infections. The median time to infection onset was 164 days (range: 2-282 days). The postoperative infectious interface keratitis developed in the early period in two cases. The mean follow-up duration was 13.4 ± 6.2 months (range: 6-26 months). Re-Descemet membrane endothelial keratoplasty was performed in two patients (25%) and therapeutic penetrating keratoplasty in four patients (50%) who did not recover with medical treatment. The final corrected distance visual acuity was 20/40 or better in five patients (62.5%).

**Conclusions:**

The diagnosis and treatment of infectious interface keratitis following Descemet membrane endothelial keratoplasty are challenging. Early surgical intervention should be preferred in the absence of response to medical treatment. Better graft survival and visual acuity can be achieved with therapeutic penetrating keratoplasty and re-Descemet membrane endothelial keratoplasty in patients with infectious interface keratitis.

## Abstract (pt)

**Objetivo:**

Avaliar o curso clínico e o manejo da ceratite infecciosa de interface após ceratoplastia endotelial da membrana de Descemet.

**Métodos:**

Um total de 352 casos submetidos a ceratoplastia endotelial da membrana de Descemet foram revisados retrospectivamente. Pacientes com ceratite infecciosa de interface foram analisados durante o acompanhamento. As análises microbiológicas, o tempo até o início da infecção, os achados clínicos, a duração do acompanhamento, o tratamento e a acuidade visual para longe corrigida pós-tratamento foram registrados.

**Resultados:**

Ceratite infecciosa de interface foi detectada em 8 olhos de 8 casos. Três patógenos fúngicos e três bacterianos foram identificados em todos os casos e receberam tratamento médico de acordo com a sensibilidade da cultura. O tratamento antifúngico foi iniciado em dois casos sem crescimento em cultura, com diagnóstico preliminar de ceratite infecciosa fúngica. Injeções antifúngicas intraestromais foram usadas em todos os casos com infecções fúngicas. O tempo médio para o início da infecção foi de 164 dias (variação: 2-282 dias). A ceratite infecciosa de interface pós-operatória desenvolveu-se no período inicial em dois casos. A duração média do acompanhamento foi de 13,4 ± 6,2 meses (variação: 6-26 meses). A ceratoplastia endotelial de membrana de Descemet foi realizada em dois casos (25%) e ceratoplastia penetrante terapêutica em quatro casos (50%) que não se recuperaram com tratamento médico. A acuidade visual para longe corrigida final foi de 20/40 ou melhor em 5/8 (62,5%) dos pacientes.

**Conclusões:**

O diagnóstico e o tratamento da ceratite infecciosa de interface após ceratoplastia endotelial da membrana de Descemet são difíceis. A intervenção cirúrgica precoce deve ser o procedimento preferido se não houver resposta ao tratamento médico. Melhor sobrevida do enxerto e melhor acuidade visual podem ser alcançadas com ceratoplastia penetrante terapêutica e ceratoplastia endotelial da membrana de Descemet em pacientes com ceratite infecciosa de interface

## INTRODUCTION

Over the past decade, anterior and posterior lamellar keratoplasties, such as deep anterior lamellar keratoplasty, Descemet stripping automated endothelial keratoplasty (DSAEK), or Descemet membrane endothelial keratoplasty (DMEK), have supplanted penetrating keratoplasty (PK) in the selective replacement of diseased corneal stroma or endothelium^(1)^. Particularly, DMEK has become the standard surgery for bullous keratopathy and Fuchs’ endothelial corneal dystrophy globally due to better visual outcomes and rapid visual rehabilitation^(2)^. Additionally, DMEK is associated with lower endothelial rejection and general complication rates compared with PK^(2)^.

Unlike PK, DMEK and other lamellar keratoplasties have a surface between the donor graft and the recipient bed termed the graft-host interface^(1)^. However, the presence of a corneal interface facilitates the development of infectious keratitis. This anatomical level is a potential space for the growth of microorganisms and consequent development of infectious keratitis. Slow multiplication of some microorganisms and the standard use of steroids following keratoplasty can mask the typical infection signs^(3)^. Because the infection occurs below the deep corneal stroma, it can also complicate the microbiological evaluation and diagnosis^(4)^. The antimicrobial drugs used in medical treatment may not achieve adequate therapeutic doses in the target tissues, resulting in decreased effectiveness^(4)^. Therefore, the diagnosis and medical treatment of infectious interface keratitis (IIK) is challenging. IIK can decrease corneal graft transparency and, if not treated, cause graft failure, endophthalmitis, and severe vision loss^(1,5,6)^. Additionally, it may lead to serious visual loss in patients with DMEK, in whom good vision outcomes are commonly expected. Unfortunately, the research on this subject is currently limited, and there are no established treatment algorithms. This study aimed to present the clinical course and management of patients with IIK following DMEK.

## METHODS

Data of 352 DMEK procedures performed between January 2014 and January 2020 were retrospectively reviewed from the medical records of patients. Patients diagnosed with IIK during the follow-up were included in the study. Conditions, such as epithelial ingrowth, noninfectious keratitis, or interface deposits that could also cause interface haze were excluded. This study was conducted according to the ethical principles of the Declaration of Helsinki and approved by the ethics committee of our hospital (protocol number 2020/514/178/16). Written informed consent was provided by the patients prior to performing DMEK.

The data collected were the patient and donor age, cause of death, death-to-preservation time, storage time in the storage solution, indication for endothelial keratoplasty (EK) (polymerase chain reaction analysis was performed for cytomegalovirus-deoxyribonucleic acid) in the aqueous humor tap of case 7 and cytomegalovirus corneal endotheliitis was diagnosed as Japan corneal endotheliitis study^(7)^ previously), endothelial cell density of the donor grafts, surgical procedure, possible predisposing factors (Table 1), time to infection onset (days), microbiological analyses, and culture results of the material obtained from the infected cornea or the changed or removed donor material, clinical findings on slit-lamp examination, treatment details, preoperative and final corrected distance vision acuity (CDVA), and any accompanying ocular and systemic disorders (Table 2).

**Table 1:** Demographic and clinical data

Patient	Patient age (years)	Donor age (years)	Donor cause of death	DTPT (h)	Storage time (days)	Donor endothelial cell density (cells/mm^2^)	Indication for endothelial keratoplasty	Surgical procedure
1	71	60	Cardiovascular disease	3	5	2,345	PBK	DMEK
2	75	57	Cardiovascular disease	1.5	1	2,450	PBK	DMEK
3	70	54	Fall from height	4	6	2,760	PBK	DMEK
4	67	65	Cardiovascular disease	3	3	2,547	FED	Triple DMEK
5	76	68	Multiple trauma	2.5	3	2,857	PBK	Scleral-fixated IOL implantation + DMEK
6	72	61	Cardiovascular disease	1.5	8	2,651	FED	Triple DMEK
7	52	59	Cardiovascular disease	8	8	2,614	CMV endotheliitis	Re-DMEK
8	70	68	Suicide	5.5	6	2,548	PBK	DMEK

CMV= cytomegalovirus; DMEK= Descemet membrane endothelial keratoplasty; DTPT= death-to-preservation time; FED= Fuchs’ endothelial dystrophy; IOL= intraocular lens; PBK= pseudophakic bullous keratopathy.

**Table 2:** The clinical course and treatment details

Patient	Preoperative CDVA (Snellen)		Possible predisposing factors of IIK	Clinical signs on slit lamb examination	Microorganism isolated from specimen		Time to infection onset (days)	Medical treatment (topical and/or systemic)	Surgical treatment		Follow-up time (months)	Final CDVA (Snellen)
1	0.016			Paracentral white interface spot infiltration		*Candida keyfr*	2	Topical amphotericin B (0.15%) Oral fluconazole 200 mg twice daily		Intrastromal voriconazole (50 µg/ml) 5 times TPK + intracameral voriconazole (50 µg/ml)	14	0.6
2	0.008	Contact lens		Paracentral 2x3 mm gray-white infiltration		*Aspergillus fumigatus*	164	Topical voriconazole (1%) IV voriconazole 200 mg twice daily		Intrastromal voriconazole (50 µg/ml) 3 times + AMT TPK + intracameral voriconazole (50 µg/ml)	16	0.05
3	0.016	Plant-based trauma		Central 3x3 mm white infiltration		*Aspergillus fumigatus*	218	Topical voriconazole (1%) IV voriconazole 200 mg twice daily		Intrastromal voriconazole (50 µg/ml) 3 times TPK + intracameral voriconazole (50 µg/ml)	13	0.8
4	0.2	Secondary graft failure TBCL Topical nepafenac		Paracentral4x3 mm epithelial defect 3x3 mm yellow infiltration 2 mm hypopion		*Pseudomonas aeruginosa*	143	Topical ceftazidime (5%) Topical gentamycin (1.4%) Oral ciprofloxacin 750 mg twice daily			19	0.6
5	0.008	Anterior chamber 1OL + multiple previous ocular surgery Epithelial ingrowth		Periferic 2x3 mm gray infiltration 1 mm hypopion		*Enterococcus faecalis*	7	Topical vancomycin (5%) Oral amoxicillin clavulanic acid (1 g) twice daily		TPK + intracameral vancomycin (1 mg/0.1 ml)	8	0.1
6	0.05	-		Periferic 2x2 mm white infiltration		*Staphylococcus epidermidis*	213	Topical vancomycin (5%) Topical gentamycin (1.4%)		-	13	0.8
7	0.05	Secondary graft failure		Paracentral 3x4 mm white infiltration		Negative	210	Topical moxifloxacin 1 % Topical voriconazole (1%) Oral voriconazole (400 mg) twice daily		Re-DMEK + intracameral voriconazole (50 µg/ml)	26	0.5
8	0.008	-		Paracentral white interface spot infiltration		Negative	141	Topical moxifloxacin (1%) Topical voriconazole (1%) Oral voriconazole (400 mg) twice daily		Re-DMEK + intracameral voriconazole (50 µg/ml)	6	0.05

AMT= amniotic membrane transplantation; CDVA= corrected distance vision acuity; DMEK= Descemet membrane endothelial keratoplasty; IIK= infectious interface keratitis; IOL= intraocular lens; TBCL= therapeutic bandage contact lens; TPK= therapeutic penetrating keratoplasty

All donor corneal buttons were provided by the University of Health Sciences Dr. Lutfi Kırdar Kartal City Hospital Eye Bank and stored in a short-term storage solution (Eusol-C^®^, Corneal Chamber; Alchimia, Ponte San Nicolò, Italy) at 4°C. Triple DMEK procedure was performed for the treatment of clinically significant coexisting cataract and Fuchs’ endothelial corneal dystrophy. Triple DMEK consists of EK following standard phacoemulsification surgery and intraocular lens implantation. DMEK, triple DMEK, and endothelial graft preparation were performed at the same stage using previously defined techniques^(2,8)^. All prepared endothelial grafts were used without delay. Subconjunctival injections of 4 mg betamethasone and 50 mg cefazolin were administered to all eyes at the end of the surgery.

Following DMEK, all eyes were treated with 0.5% moxifloxacin hydrochloride (Vigamox; Alcon Pharma GmbH, Freiburg, Germany) and 0.1% dexamethasone (Maxidex; Alcon Pharma GmbH) five times daily. Treatment with the topical antibiotic was discontinued after 10 days. Three months after surgery, dexamethasone was replaced with 0.5% loteprednol etabonate (Lotemax; Bausch + Lomb, Bridgewater, NJ, USA) four times daily. The topical treatment with steroid was gradually tapered based on the clinical outcome of each patient.

**Figure 1:**
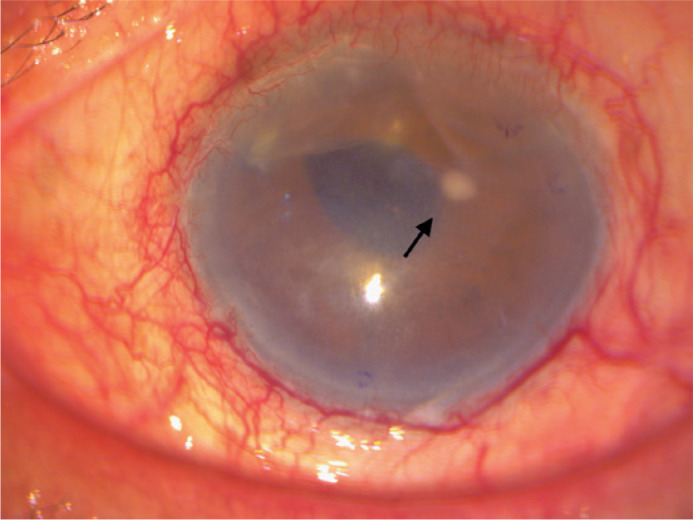
Early fungal IK after DMEK. Patient 1: photograph 2 days after DMEK showing a single white interface spot (arrow).

The diagnosis of IIK was based on slit-lamp examination signs of infectious keratitis at the graft level during follow-up. For example, a fungal interface keratitis (IK) showed small white corneal interface spots with minimal anterior chamber inflammation (Figure 1). Worsening of the infection is manifested by an increase in the infiltration size and assuming less defines its limits accompanied by stromal edema. Using forceps, microbiological samples were obtained from the deep-seated infiltrate associated with the epithelial defect. In cases of intact epithelium, the anterior chamber was accessed through a side port incision, and approximately 1 mm of the infected posterior lamellar was incised using micro-vitrectomy scissors under viscoelastic material (sodium hyaluronate 1.4%; bio-hyaluronic acid EV; Biotechnology, India) as previously described^(9)^. The samples were sent to the laboratory for microbiological examinations. According to the culture sensitivity results or clinical findings in cases with negative cultures, topical fortified antifungal and/or antibacterial treatment was started at an initial loading dose of eye drops every 5 min for the first 30 min, followed by eye drops hourly for 48 h. The frequency of the fortified drops was subsequently decreased to every 3 h. Systemic antifungal and/or antibacterial drugs were added according to the depth, size, and clinical progression of keratitis. In cases with fungal IK, intrastromal antifungal injections at 72-h intervals were administered according to the depth and size of the infiltration. In cases in which a lack of response to the medical treatment was observed, therapeutic penetrating keratoplasty (TPK) and re-DMEK were scheduled.

DMEK= Descemet membrane endothelial keratoplasty; IK= interface keratitis

TPK and re-DMEK, including the whole endothelial graft, were performed using a donor cornea or endothelial graft 0.5 mm wider than the receiver’s infected cornea or endothelial graft. Anterior chamber irrigation was performed using antifungal or antibacterial drugs. The removed or replaced tissues were sent to the laboratory for microbiological analyses.

Samples were examined for the presence of aerobic, anaerobic, and fungal microorganisms. Thioglycolate broth was used for the isolation of organisms from the corneal culture. The sample was incubated in thioglycolate broth at 35°C for 24 h and plated onto media (5% sheep blood agar, chocolate agar, Brucella agar, and Sabouraud dextrose agar). All plates were incubated in 5%-7% CO_2_ at 35°C for 72 h. The plates were evaluated daily for the growth of microorganisms, and antimicrobial susceptibility tests were performed following the detection of growth (VITEK 2^®^ Compact Systems; BioMerieux, France).

The administration of fortified antifungal/antibacterial drops used preoperatively (Table 2) was continued every 2 h during the initial 48 h of the postoperative period. Subsequently, treatment was tapered to every 3 h for an average of 3 weeks following TPK or re-DMEK. In case of fungal IK, topical cyclosporin 0.1% four times daily was added to this treatment regimen during the first postoperative week. In the absence of IK recurrence, topical cyclosporin was replaced with topical dexamethasone 0.1% twice daily. In case of bacterial IK, administration of topical dexamethasone 0.1% at least 3-5 times daily was initiated in the immediate postoperative period, with the dose adjusted according to the condition of each patient. Treatment with dexamethasone was subsequently replaced with loteprednol etabonate 0.5%, which was gradually tapered and eventually discontinued.

Treatment success was defined as infection control without anterior chamber inflammation or interface infiltration. During follow-up, the preoperative and final CDVA were evaluated using the Snellen chart.

## RESULTS

**Figure 2:**
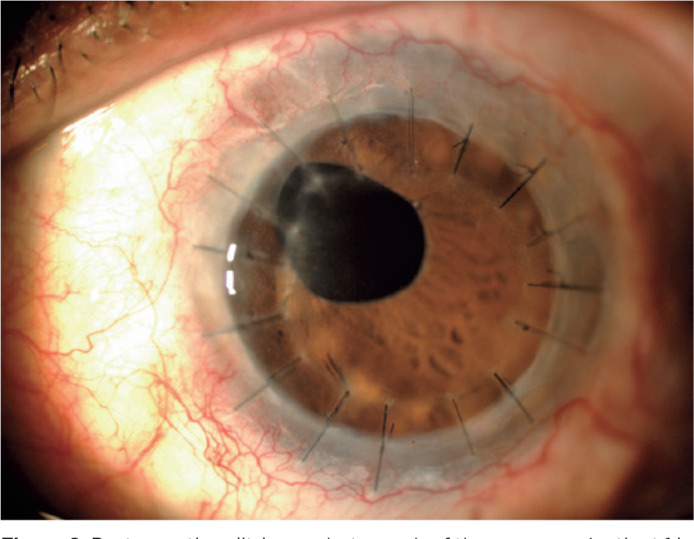
Postoperative slit-lamp photograph of the same eye (patient 1 in Table 2) a few months after therapeutic penetrating keratoplasty showing a clear cornea.

Eight eyes of eight patients who underwent DMEK and developed IIK during the follow-up were analyzed. The mean donor age was 61.7 ± 5.48 years (range: 54-68 years), and the commonest cause of death was cardiovascular disease. The mean death-to-preservation time was 3.62 ± 2.19 h (range: 1.5-8 h), and the mean storage time was 5 ± 2.5 days (range: 1-8 days). The mean donor endothelial cell density was 2,596.5 ± 163.6 cells/mm^2^ (range: 2,345-2,760 cells/mm^2^). The demographic and clinical data of the patients and the donors are presented in Table 1. The mean age of patients was 69.1 ± 7.49 years (range: 52-76 years), and the median time to clinical infection was 164 days (range: 2-218 days) after DMEK. IIK findings were noted on postoperative days 2 and 7 in patients 1 (Figure 1) and 5, respectively, and subsequently in the remaining patients. Microbiological analyses and culture results revealed fungal infection in three patients (37.5%) (cases 1-3) and bacterial infection in three patients (37.5%) (patients 4-6) (Table 2). Antifungal treatment was initiated with a preliminary diagnosis of fungal IK in two patients (25%) in which no growth was detected on culture. Therefore, five patients (62.5%) received antifungal treatment. Multiple intrastromal antifungal injections were administered to the three patients (37.5%) (patients 1-3) with fungal growth detected on culture. Microbiological analyses and culture results were negative in patients 7 and 8. In patient 7, polymerase chain reaction analyses, performed twice for the aqueous humor tap, were negative for cytomegalovirus-deoxyribonucleic acid. Despite the accompanying glaucoma findings in patient 8, endothelial decompensation was not considered clinically. According to the microbiological findings of graft infection, a preliminary diagnosis of IIK was established in these patients, and empirical antibiotic therapy was initiated as described in the Methods section. Re-DMEK was performed for graft replacement in two patients (25%) (patients 7 and 8), and TPK was performed in four patients (50%) (patients 1, 2, 3, and 5) (Figure 2) because of the lack of improvement following medical treatment. Recurrent infection or endophthalmitis secondary to IIK was not observed in any patient. The final CDVA was 20/40 or better in five patients (62.5%). All patients were followed up for a mean duration of 13.4 ± 6.2 months (range: 6-26 months). The clinical course and treatment details are presented in table 2.

## DISCUSSION

IIK is a rare but critical complication of DMEK, which develops at the graft-host interface created during lamellar keratoplasty^(3,10)^. A high level of suspicion is required for the preliminary diagnosis of IIK because infection usually manifests with minimal inflammatory signs and symptoms. Initially findings of IIK on slit-lamp examination include a typically clear cornea, or single/multiple gray-white infiltrates located at the graft-host interface^(10)^. Therefore, early diagnosis and prompt treatment of IIK are important. Some studies have reported the occurrence of IIK after anterior lamellar keratoplasty and DSAEK^(3,5,10-12)^. However, there are only a few case reports of IIK following DMEK and only one case series of fungal IK^(10,11)^. To our knowledge, this is the first large series study of both fungal and bacterial IK following DMEK.

A report published by the Eye Bank Association of America revealed a higher frequency of fungal infections following EK (0.022%) than after PK (0.012%)^(13)^. Fungal agents are the commonest pathogens associated with the development of IIK following keratoplasty^(10,11)^. Augustin et al. reported a fungal IK rate of 0.15% in their series^(14)^. In the present study, fungal culture was positive in three patients (37.5%); of note, the clinical course was suspicious for fungal keratitis in two patients (25%) with a negative culture result. Therefore, 62.5% of our patients required antifungal treatment. Nahum et al. reported an IK rate of 0.92% following DSAEK^(3)^. However, in contrast to the present study and data available in the literature, the causative agent was bacterial in 0.53% and fungal in 0.39% of the patients^(3)^. *Candida* spp. has been reported as the commonest fungal pathogen linked to IIK following EK^(10-12)^. Augustin et al. reported that *Candida* spp. was the causative agent in all cases of fungal IK following DMEK^(14)^. Regarding our cases of fungal IK, *Candida keyfr* and *Aspergillus fumigatus* grew in one and two cases, respectively. Other studies have demonstrated that *Aspergillus* was the cause of IIK following anterior lamellar keratoplasty^(15,16)^, DSAEK^(9)^, laser in-situ keratomileusis^(17)^, and intracorneal ring implantation^(18)^. Contact lens and trauma were the risk factors in these cases, and rapid corneal melting has also been reported^(17,18)^. Our patients in whom *Aspergillus fumigatus* growth was detected had the same risk factors, and corneal melting was present in patient 2. In this study, early TPK was performed in all IK cases with fungal growth in culture, and the grafts were clear on follow-up. Augustin et al. successfully treated six fungal IIK cases, using TPK and re-DMEK in 66% and 33% of them, respectively^(14)^. Additionally, Tsui et al. eradicated the infection in eyes with fungal IK developing after DSAEK using TPK and re-DSAEK in 76% and 5%, respectively^(12)^. Our study has demonstrated that the final successful treatment in most IK cases with a fungal cause is graft replacement at the early stage, and TPK is frequently required^(3,10,11,14,19)^.

Intrastromal antifungal injections can be added to the medical treatment for persistent fungal IK^(4,20)^. We injected intrastromal voriconazole to the three cases with a fungal microorganism detected in the culture (patients 1-3). This procedure can either increase the effectiveness of medical treatment or decrease the infiltration area in the graft replacement procedure. It can also provide some time to prepare the receiver cornea for TPK and/or prevent the recurrence of infection by decreasing the infection load prior to TPK^(20)^. Based on this evidence in the literature and our clinic observations, we propose that intrastromal treatment plays a role in the prevention of infection recurrence after TPK and in maintaining a clear graft during follow-up in our three cases presented above. However, it is possible for the thin graft to become perforated and the clinical course to change from keratitis to endophthalmitis^(20)^. This possibility should not be overlooked, and the procedure should be performed carefully and only in suitable cases.

The IIK onset time in our study demonstrated a wide range (2-282 days). Our two early-onset cases with *Candida* and *Enterococcus* growth (patients 1 and 5) exhibited a rapid and aggressive course. The course of most early-onset IK cases reported after EK has been sudden and aggressive^(3,14)^. We considered contamination of the donor or storage solution as the source of the infection in these cases, as reported in the literature. However, it was not possible to prove this, as we were unable to culture the donor rim or storage solution^(11,21)^. Corneas obtained from donors after their death due to cardiovascular causes have been associated with an increased risk of fungal contamination after keratoplasty^(22)^. Therefore, the cause of death of the donor in patient 1 may have facilitated the development of fungal IIK. The risk factor for patient 2 could be the multiple ocular surgeries performed prior to triple DMEK, as this has been reported as the major risk factor for *Enterococcus* keratitis in the literature^(23)^. In their case report, Beckman et al. emphasized that IIK of donor origin is not exclusively observed in the early period. They reported that despite the positive donor culture and prophylactic treatment of their patients, fungal IIK developed during the first year after DSEK^(24)^. Both earlyand late-onset IIK can be of donor origin. Therefore, it is important to perform donor rim culture to plan the appropriate treatment regimen^(10,11,14,24)^.

Graft failure was the common feature of late-onset cases (patients 4 and 7). The therapeutic bandage contact lens may have facilitated the development of IK of *Pseudomonas* origin in patient 4, in whom epitheliopathy accompanied graft failure; moreover, the use of 0.1% topical nepafenac could have accelerated the epitheliopathy^(25,26)^. IIK develops at the graft-host interface; however, in these patients, the infection may have facilitated its progression at the interface, possibly with an epithelial defect. In their IK case of *Nocardia* origin, Srirampur et al. reported a clinical course and etiopathogenesis similar to those of the present patient 4^(27)^. Therefore, it is important to monitor the patients for ocular surface dysfunction, as this condition can accompany post-DMEK graft failure.

In this series, TPK, re-DMEK, and medical treatment alone were employed in 50%, 25%, and 25% of all cases, respectively. These results are similar to those reported in a IIK review involving 62 cases: TPK in 62.9%, lamellar keratoplasty alone in 12.9%, and medical treatment alone in 24.2%^(11)^. These results indicate that surgical treatment should be preferred in IIK when the desired outcome is not achieved with medical treatment.

A limitation of this study was that we were unable to obtain donor rim cultures in contrast to what has been previously reported in the literature. The lack of anterior segment optic coherence tomography or confocal microscopy findings that would have supported our conclusions is another limitation. However, our study is valuable as it is the first series presenting fungal and bacterial IK after DMEK. Furthermore, it also included a large series of patients. In addition, this is the first study to report IK of *Aspergillus, Enterococcus*, and *Pseudomonas* origin following DMEK.

In conclusion, careful clinical examination and microbiological evaluation are the main principles for the management of IIK. Although it is possible to partially eliminate the infection with medical and intrastromal treatment, re-DMEK or TPK is required in most of these patients. Through these surgical approaches, it is possible to ensure anatomical, clinical, and visual improvement in these patients.
